# Crescentic glomerulonephritis with dual positive anti-GBM and C-ANCA/PR3 antibodies 

**DOI:** 10.5414/CNCS108666

**Published:** 2016-04-29

**Authors:** Bolanle A. Omotoso, Helen P. Cathro, Rasheed A. Balogun

**Affiliations:** 1 Department of Internal Medicine, Division of Nephrology,; 2 Department of Pathology, University of Virginia, Charlottesville, VA, USA, and; 3 Renal Unit, Department of Medicine, Obafemi Awolowo University Teaching Hospital, Ile Ife, Osun State, Nigeria

**Keywords:** crescentic GN, antiglomerular basement membrane antibody disease, ANCA-associated vasculitis

## Abstract

Antiglomerular basement membrane (anti-GBM) antibodies are more often accompanied by myeloperoxidase antineutrophil cytoplasmic antibody (MPO-ANCA) than by proteinase 3 antineutrophil cytoplasmic antibody (PR3-ANCA). Both disease processes can affect the kidneys and/or the lungs. Patients with dual positive disease may have an atypical presentation which may delay diagnosis and treatment. Here we report a case of crescentic glomerulonephritis associated with positive PR3-ANCA and anti-GBM antibodies who underwent both lung and kidney biopsies.

## Introduction 

About one in three patients with anti-GBM antibodies will also test positive for ANCA. Patients with dual positivity are more likely to have P-ANCA pattern and a higher specificity for MPO than PR3 [1, 2, 3]. The presentation may be atypical, delaying diagnosis and treatment, resulting in a worse clinical outcome. ANCA-associated disease relapses are common in this group of patients [4]. Thus, patients with suspected pulmonary renal syndrome should be identified early, checked for anti-GBM and ANCA antibodies, and followed closely for relapse. Here we report a 78-year-old woman who presented with an unusual combination of anti-GBM nephritis and granulomatosis with polyangiitis with limited lung disease that was initially treated as a case of multifocal pneumonia. 

## Case report 

A 78-year-old Caucasian woman presented with a 3-week history of cough, intermittent hemoptysis and epistaxis, weight loss, pleuritic chest pain, malaise, and arthralgia, but no joint pain or swelling. The primary physician had made a diagnosis of multifocal pneumonia based on symptoms and chest radiographic findings, and the patient was placed on a 2-week course of antibiotics (amoxicillin and azithromycin). At that time, the SCr was 0.9 mg/dL. The patient returned to the emergency department 2 weeks later with worsening cough, chest pain radiating to the shoulders, reduction in urine output, worsening malaise, arthralgia, anorexia, nausea, and vomiting. 

Examination revealed an elderly woman, not in obvious respiratory distress, pale, not cyanosed, with no pitting pedal edema. There was no skin rash or mucosal ulceration. The sinuses were not tender. Pulse was 99 beats/min and regular, blood pressure was 114/64 mmHg. The precordium was normoactive, and the 1^st^ and 2^nd^ heart sounds were heard. The patient was tachypneic, with a RR 23 cycles/min. Oxygen saturation was 98% at room air and coarse breath sounds were heard in all the lung fields. 

A basic metabolic panel revealed: sodium 128 mmol/L, potassium 3.8 mmol/L, bicarbonate 11 mmol/L (23 – 31 mmol/L), BUN 82 mg/dL (9.8 – 20 mg/dL), SCr 8.3 mg/dL (0.6 – 1.1 mg/dL), chloride 99 mmol/L, calcium 9.1 mg/dL (8.5 – 10.5 mg/dL), and anion gap 20 (5 – 15). SCr was 0.8 mg/dL and 1.2 mg/dL 2 and 1 weeks previously, respectively. During admission SCr increased to 9.4 mg/dL within 24 hours. 

Urine dipstick was positive for blood and protein. Fractional excretion of sodium (FE Na) and FE urea were 7.5% and 63.6%, respectively. Urinary protein Cr ratio was 2.7. 

Urine microscopy showed numerous eumorphic red blood cells (RBC), some dysmorphic RBC, a few white blood cells (WBC) and few granular casts. WBC count at admission was 10,000/cm^3^, and hemoglobin concentration was 7.7 g/dL. 

Renal ultrasound revealed normal sized kidneys with increased echogenicity and no hydronephrosis. Chest radiograph showed persistence of a mass-like opacity in the right upper lobe similar to that seen on chest X-ray on the outpatient visit. Computerized tomography (CT) revealed multifocal spiculated nodules and masses within both lungs; the largest measuring ~ 3.3 × 4.8 cm in the right upper lobe, thought to represent an obstructing mass with resultant adjacent atelectasis. Further workup revealed normal C3 and C4 levels, negative ANA, ASO titers, and rheumatoid factor. C-ANCA was positive directed against PR3; titer > 8 Antibody Index (AI) (< 1.0 AI). P-ANCA was negative. Her anti-GBM IgG antibody was also positive > 8 AI (< 1.0AI), and C-reactive protein was elevated 24.5 mg/dL (< 0.5 mg/dL). Serology for hepatitis B, hepatitis C, and HIV were all negative. Serum and urinary protein electrophoresis were unremarkable. Renal and CT-guided lung biopsies were performed. 

### Renal biopsy 

**Figure 1.**
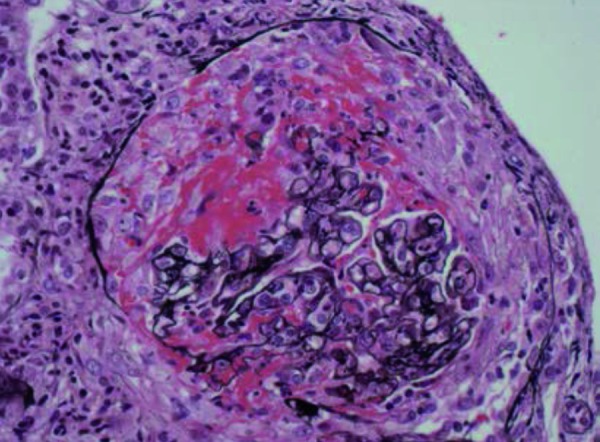
Glomerulus showing a cellular crescent with fibrinoid necrosis (Jones methenamine silver stain × 200).

**Figure 2.**
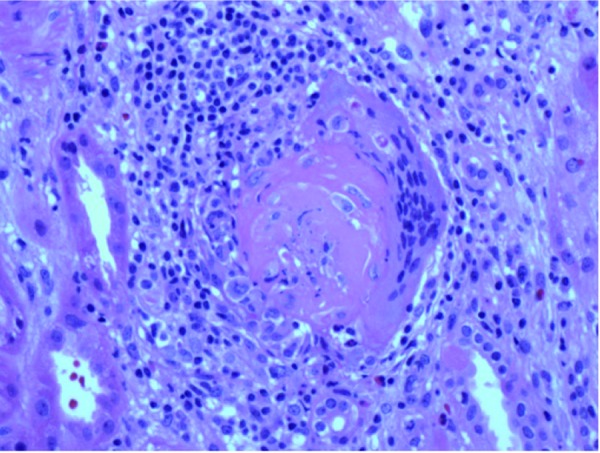
Obsolescent glomerulus with adjacent multinucleated giant cell (H & E × 200).

Light microscopy revealed 4 corticomedullary cores with 28 glomeruli, 5 of which were obsolescent. 16 glomeruli demonstrated cellular crescents with marked fibrinoid necrosis (Figure 1). Obliteration of Bowman’s capsules and periglomerular giant cells were noted in a few glomeruli (Figure 2). A marked acute and chronic interstitial infiltrate was present. Numerous red cell casts were noted. Mild tubular atrophy was accompanied by mild interstitial fibrosis. Arteries were sclerotic with no inflammation. A Congo red stain was negative. 

**Figure 3.**
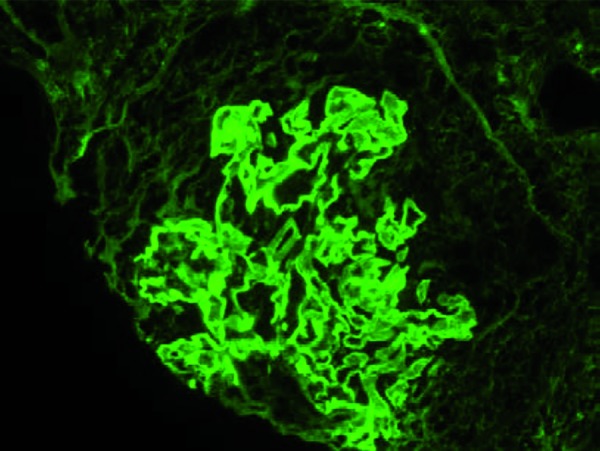
Bright linear IgG staining of the glomerular capillary loops on immunofluorescence.

Immunofluorescence was performed on 5 glomeruli, all of which had cellular crescents. Bright capillary loop staining was seen with antisera specific for IgG (2+; scale trace through 3+), C3 (1+), and κ and λ light chains (both 2+) (Figure 3). Fibrinogen stained the crescents. No tubular basement membrane staining was seen. 

**Figure 4.**
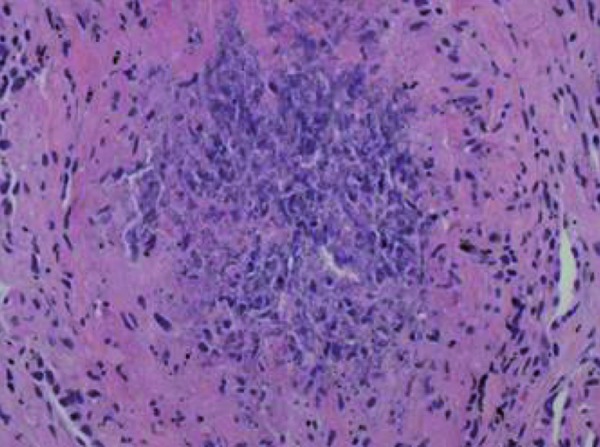
Necrotizing arteritis with transmural inflammation in the lung instertitium (H & E × 200).

**Figure 5.**
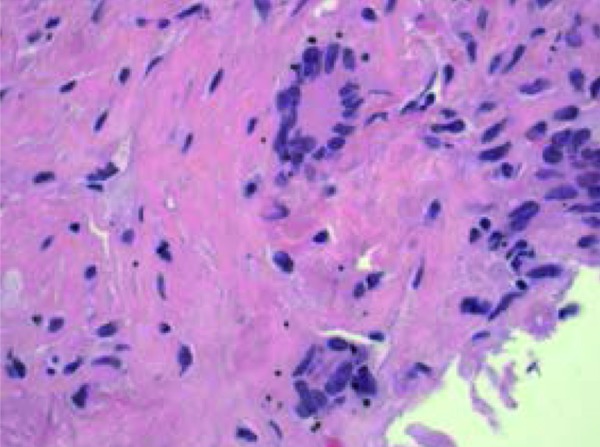
Multinucleated giant cells in the lung (H & E × 400).

Ultrastructural examination of single glomerulus demonstrated diffuse fibrinoid necrosis and marked endocapillary hypercellularity with numerous breaks in the capillary loop basement membrane. There were no immune complex-type electron dense deposits or tubuloreticular inclusions. Proximal convoluted tubular and peritubular capillary profiles were unremarkable. The CT-guided lung biopsy revealed multiple cores with necrotizing granulomatous inflammation and focal vasculitis with associated multinucleated giant cells (Figure 4, Figure 5). A single fragment showed hemorrhage with focal organization. An assessment of anti-GBM antibody nephritis with 57% active crescents and ANCA-positive vasculitis presenting predominantly with extra-renal manifestations (granulomatosis with polyangiitis) was made. 

Dialysis was initiated. The patient was immediately commenced on high-dose IV methylprednisolone 500 mg daily for 3 days, followed by high-dose oral prednisolone 60 mg. The patient also received 4 doses of IV rituximab 375 mg/m^2^ weekly as part of induction therapy (as per the RAVE trial), and 6 sessions of alternate day therapeutic plasma exchange (TPE) [5]. The anti-GBM antibodies disappeared and the PR3 ANCA dropped to 1.8 AI in 10 weeks. The patient is still dialysis-dependent 3 months later, but has had full resolution of respiratory and other systemic symptoms. 

## Discussion 

Anti-GBM disease is a rare, potentially life-threatening disease with a prevalence < 1 case/million population per year [5]. It is characterized by rapidly progressive glomerulonephritis, pulmonary hemorrhage, and elevated titers of anti-GBM antibody. The pathological hallmark is the formation of antibody against glomerular basement membrane epitopes in the α3 chain of type IV collagen and is characterized by a linear immunoglobin G (IgG) deposition along the GBM on immunofluorescence microscopy. 

Though pulmonary renal syndrome is the most common presentation, ~ 1/3 of patients present with isolated GN (anti-GBM antibody nephritis), especially the elderly. Isolated pulmonary hemorrhage is an uncommon feature of anti-GBM nephritis. Constitutional symptoms such as rash, fever, arthralgia, malaise, and weight loss are even less common. The presence of such symptoms suggests that the patient may have a concurrent vasculitis [6]. 

Approximately 1/3 of patients with anti-GBM disease have ANCA antibodies usually P-ANCA [7, 8, 9]. Cases with dual positivity usually present at an age of 50 – 80 years, which corresponds to the typical age range of patients with ANCA-associated vasculitis. In contrast, pure anti-GBM disease has a bimodal distribution, occurring in men in their 20s and women older than 80 years [5]. Lockwood et al. [10] found that ANCA may be detected together with anti-GBM antibodies; however, the relationship with disease expression was uncertain. O’Donoghue et al. [1, 11] were the first to bring attention on the presence of ANCA in patients with anti-GBM disease. Subsequently, prospective studies with large series of patients were performed (Table 1). These authors concluded that clinical and pathologic data collected from patients with both antibodies suggested the coexistence of a systemic vasculitis. 

The reason for concurrent ANCA and anti-GBM antibodies in this group of patients is unclear. There appears to be no structural relationship between ANCA and the α3 non-collagenous domain of type IV collagen or a direct cross-reactivity between these two antibodies. This supports the concept that the two diseases are separate pathological entities [3, 12]. It was speculated that immune dysregulation triggers the development of concurrent ANCA and anti-GBM disease [7]. 

Another hypothesis for their co-existence is that the ANCA vasculitis or the underlying process causing the vasculitis itself injures the GBM, uncovering hidden antigens on the GBM that leads to auto-immunogenicity, thus initiating development of anti-GBM antibody [13]. 

The dual positive patient reported here belongs to a smaller group of patients who have C-ANCA with PR3 specificity since MPO-ANCA is more frequent than PR3-ANCA in patients who are also positive for anti-GBM [2, 3, 9]. 

A possible explanation for the co-existence of both types of antibodies in our patient might be that the anti-GBM antibody production started after the injury to the GBM by ANCA since she initially presented with cough, and constitutional symptoms of vasculitis, and a lung biopsy was suggestive of granulomatosis with polyangiitis. However, if this were the case, one would expect the renal biopsy to show both active and chronic lesions, in keeping with the typically frequent relapses in ANCA-associated disease [14]. Moreover, ANCA vasculitis has a clinically indolent course and as a result, the duration of disease from the first clinical sign of any organ involvement prior to biopsy is substantially longer in ANCA vasculitis than in anti-GBM antibody-mediated disease [15, 16, 17]. The renal biopsy in this case revealed only active lesions (cellular crescents) at the same stage of evolution. Thus, it is possible that the two diseases occurred sequentially in our patient. Her initial presenting symptoms; pulmonary imaging and lung histology findings were more suggestive of limited granulomatosis with polyangitis. The subsequent development of anti-GBM nephritis is supported by the presence of active crescents at the same stage of evolution. 

Further studies on the role of ANCAs in the pathogenesis of dual-positive disease will be necessary to unravel the mechanisms of damage. 

The treatment for dialysis-independent dual-positive disease is usually the same as that for isolated anti-GBM nephritis. High-dose steroids (1 mg/kg), oral cyclophosphamide (2 mg/kg) daily or on alternate days, and therapeutic plasma exchange (TPE) to remove circulating anti-GBM antibodies until levels are undetectable usually lasts 2 – 4 weeks. Immunosuppressive treatment typically lasts for 3 – 6 months. Rituximab has been used successfully in refractory disease [18, 19]. 

For dialysis-dependent dual-positive disease, the management would be that recommended for dialysis-dependent ANCA vasculitis patients: oral or IV cyclophosphamide/IV rituximab, IV pulse steroids followed by high-dose oral steroids; dialysis and TPE [20, 21, 22, 23]. IV rituximab is not inferior to cyclophosphamide treatment for induction of remission, but may be superior in relapsing disease [21]. 

Kidney transplantation is an option for renal replacement therapy for end-stage renal disease, but most centers delay the procedure until the patient has been negative for anti-GBM antibodies for at least 12 months. 

In our patient, plasma exchange and immunosuppressive treatment led to the resolution of pulmonary hemorrhage and other systemic symptoms of vasculitis. Despite this aggressive treatment, the patient has yet to recover renal function. This is not unexpected as 1-year renal recovery is said to be absent (0%) in dual-positive patients with SCr > 5.7 mg/dL (500 mmol/L) and/or dialysis-dependence at presentation [7, 14]. Though anti-GBM nephritis is a monophasic disease which rarely recurs, it is also important to note that recurrence of disease activity with ANCA antibodies is possible [1, 14, 16, 24, 25]. The risk is high in patients with persistently elevated ANCA after resolution of initial symptoms [20, 25]. Serial ANCA antibody testing, though controversial, may be useful. Some patients may exhibit a consistent and recurring pattern of rising ANCA titers preceding each clinical relapse of disease (especially anti-PR3-positive patients) [20, 26]. Unlike isolated anti-GBM disease patients who do not require chronic immunosuppression, such patients require long-term maintenance immunosuppression [20, 26]. 

## Acknowledgments 

Dr. Omotoso is a nephrology trainee at the Obafemi Awolowo University Teaching Hospital, Ile-Ife, Nigeria. She is currently an International Society of Nephrology fellow under the mentorship of Dr. Balogun. 

## Conflict of interest 

None. 

**Table 1.** Reported series of ANCA- and anti-GBM-associated disease.

Author (Ref)	No. of AGBM cases	No. of AGBMA & ANCA + ve cases	MPO	PR3	Undetermined antigen specificity	P-ANCA	C-ANCA	Negative
O’ Donoghue [12]	12	3	0	3	–	0	3	–
Jayne et al. [2]	67		N.A.	N.A.	N.A.	7	11	2 were negative for both P- and C-ANCA on IIF
Bosch et al. [1]	37	12	11	1	–	11 (all had anti- MPO antibody specificity	1	–
Weber et al. [10]	23	8	7	1	–	0	1 (PR3 specificity)	7 negative for P-ANCA on IIF (but all 7 were + ve for MPO)
Bonsib et al. [24]	12	6	2	2	2	4 (2 MPO, 2 undetermined antigen specificity	2 (PR3 specificity)	–
Short et al. [3]	160	34	25	3	6	19 (17 MPO, 1 PR3, 1 undetermined antigen specificity)	15 (8 MPO, 2 PR3, 5 undetermined antigen specificity)	–
Lindič et al. [14]	48	8 (out of 30 screened for ANCA)	8	–	–	NA	NA	NA

AGBM = antiglomerular basement membrane; AGBMA = antiglomerular basement membrane; P-ANCA = perinuclear antineutrophil cytoplasmic antibody; C-ANCA = cytoplasmic pattern of antineutrophil cytoplasmic antibody; MPO = antimyeloperoxidase specificity; PR3 = anti proteinase 3 specificity; undetermined antigen specificity = MPO-negative and PR3-negative; IIF = indirect immunofluorescence microscopy; ELISA = enzyme-linked immunosorbent assay; N.A. = antigen specificity (by ELISA method) was not reported in the study.
